# Parafoveolar retinoblastoma regression with foveal preservation following intra-arterial chemotherapy documented on hand-held optical coherence tomography in a newborn

**DOI:** 10.1186/s40942-017-0098-3

**Published:** 2017-11-13

**Authors:** Vera Yarovaya, Kareem Sioufi, Carol L. Shields

**Affiliations:** Ocular Oncology Service, Wills Eye Hospital, Thomas Jefferson University, 840 Walnut Street, Suite 1440, 14th Floor, Philadelphia, PA 19107 USA

**Keywords:** Eye, Retina, Retinoblastoma, Optical coherence tomography (OCT), Intra-arterial chemotherapy, Foveola, Fovea

## Abstract

**Background:**

Optical coherence tomography (OCT) has become an invaluable tool in retinoblastoma management, providing submillimeter visualization of tumor control following treatment. Herein, we document OCT-detection of a subtle tumor recurrence, allowing early intervention and achieving foveal microanatomy preservation.

**Case presentation:**

A 3-week-old girl was diagnosed with bilateral familial retinoblastoma, classified as group D in the right eye (OD) and group B in the left eye (OS), and treated with intravenous chemoreduction. At 6-months follow-up, the right eye was under control, but the left eye revealed a subtle juxtafoveal tumor recurrence, documented on handheld OCT (HH-OCT) and measuring 2750 µm in diameter and 792 µm in thickness. Treatment with intraarterial chemotherapy (IAC) using 1 cycle of melphalan 5 mg was performed and complete tumor control was achieved, leaving a flat, concave scar 663 µm from the intact foveola and measuring 2750 µm in diameter and 120 µm in thickness. Foveal microanatomy OS was preserved on HH-OCT. The findings remained stable at 2 years following IAC.

**Conclusions:**

HH-OCT is an important tool in retinoblastoma management. In this case, HH-OCT allowed for early detection of retinoblastoma recurrence, before foveal invasion. Following treatment with IAC, complete tumor regression was noted and foveal microanatomy remained intact.

## Background

Optical coherence tomography (OCT) has become an invaluable tool for in vivo evaluation of the microstructure of the retina and choroid. This technology employs light scatter to create a 2-dimensional cross-sectional image of the human fundus, initially described by Huang et al. [1]. With advancements in technology and development of spectral-domain (SD-OCT), high resolution in vivo evaluation of retinal microanatomy in healthy and diseased eyes now approaches resolution of tissue histology. The subspecialty of ocular oncology has incorporated SD-OCT into practice for the study of the structure of various retinal, choroidal, optic disc, and scleral tumors and their relationship to other structures [2]. Handheld OCT (HH-OCT), a portable, SD-OCT unit that can be used in the operating room, has become a crucial diagnostic tool in pediatric retinal diseases as it provides accurate localization of disease processes and is completely noninvasive [3]. HH-OCT is also used in the management of retinoblastoma, providing submillimeter visualization of tumor development and regression, far beyond the detection by ophthalmoscopy. In addition, this technology allows for detection of invisible tumors [4, 5], and monitoring of foveal microanatomy for visual prognostication [6].

Herein, we report the case of a 3-week-old girl with bilateral familial retinoblastoma treated with systemic chemoreduction. Tumor recurrence was confirmed on HH-OCT and was subsequently controlled with intraarterial chemotherapy (IAC). Documentation with HH-OCT allowed accurate monitoring of the tumor as well as the foveola.

## Case presentation

A 3-week-old girl, with a family history of maternal unilateral retinoblastoma, presented for evaluation of leukocoria right eye (OD). On examination, visual acuity was fix and follow in both eyes (OU) and intraocular pressures were normal OU. External examination documented obvious leukocoria OD. Fundus evaluation OD revealed a white macular tumor measuring 16.0 mm in largest basal dimension and 6.1 mm in thickness, and with overlying mild vitreous seeding and surrounding extensive serous retinal detachment. Fundus evaluation of the left eye (OS) detected a solitary mass measuring 2.0 mm in basal dimension and 1.0 mm in thickness, located within 2.0 mm from the foveola. A diagnosis of bilateral familial retinoblastoma, group D OD and group B OS, was rendered and treatment with intravenous chemoreduction (CRD) using vincristine, etoposide, and carboplatin was initiated. Following therapy with individual tumor consolidation, all retinoblastomas were regressed.

**Fig. 1:**
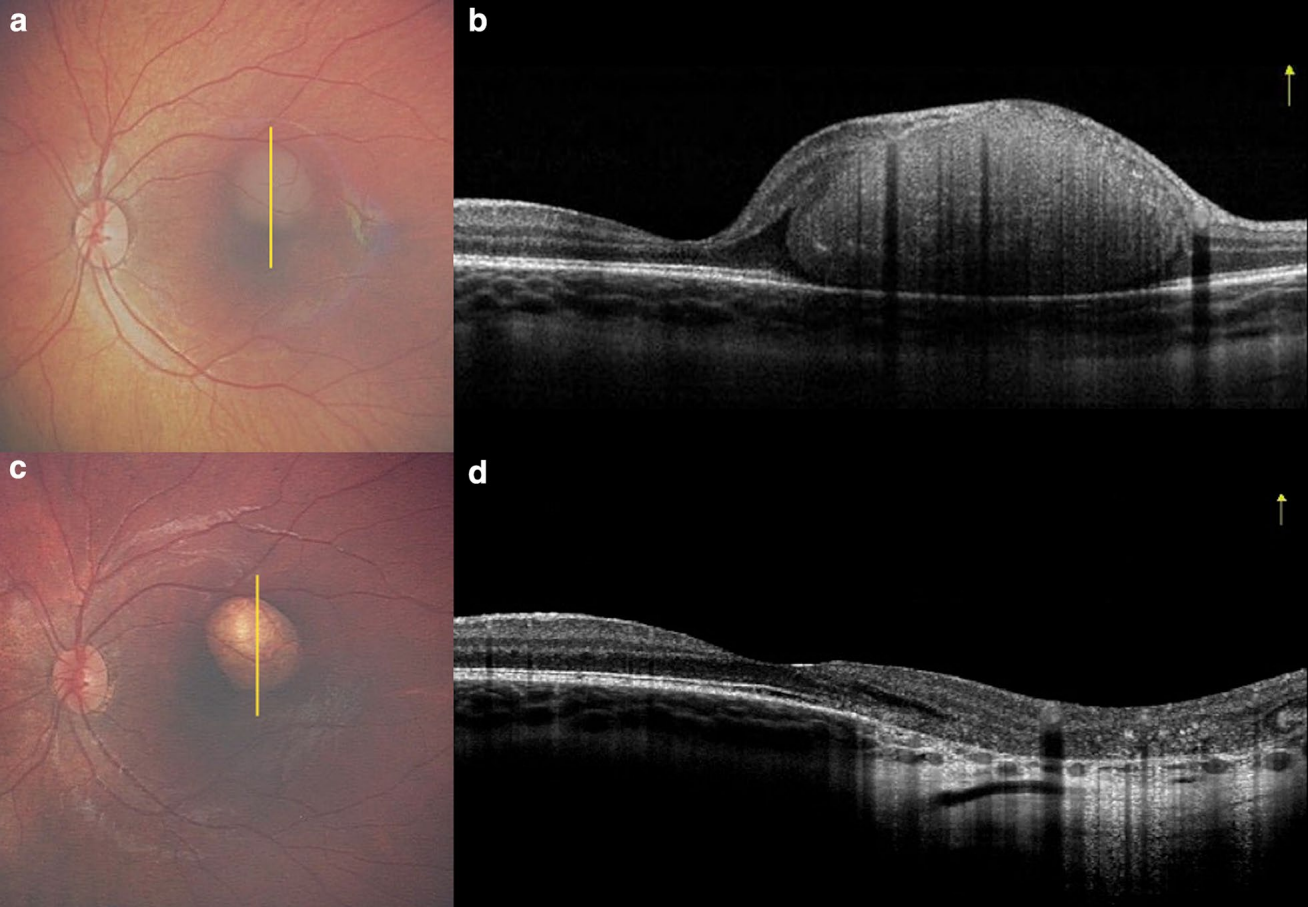
Documentation of retinoblastoma control using spectral domain hand-held optical coherence tomography (HH-OCT). Following 6 cycles of intravenous chemotherapy for bilateral retinoblastoma, the left eye demonstrated small dome-shaped, outer retinal tumor recurrence (**a**) of 2750 µm in basal dimension and 792 µm thickness in the parafoveolar region, documented on HH-OCT (**b**) and with adjacent shallow subretinal fluid. The mass was 615 µm from the foveola. Following 1 cycle of intraarterial chemotherapy, the tumor showed complete regression (**c**) and HH-OCT (**d**) documented a concave scar of 120 µm in thickness and located 663 µm from foveola. There was OCT light transmission through atrophic retinal pigment epithelial accentuating the underlying thin choroid. The foveola remained intact

At 6-months follow-up, the right eye remained under control, but the left eye revealed a subtle recurrence of the juxtafoveal tumor (Fig. 1a) and HH-OCT (iVue Optovue, Fremont, CA) revealed an intact macula with adjacent tumor recurrence (Fig. 1b), measuring 2750 µm in diameter and 792 µm in thickness. The recurrence was 615 µm from the foveola. Treatment with intraarterial chemotherapy (IAC) using Melphalan 5 mg was performed and complete tumor control was achieved with 1 cycle (Fig. 1c), leaving a concave scar of 2750 µm in diameter and 120 µm in thickness, located 663 µm from the foveola. In addition, there was underlying choroidal thinning and preservation of the foveal microanatomy documented by HH-OCT OS (Fig. 1d). The findings remained stable on last follow-up at 2 years following IAC.

## Discussion and conclusions

Chemotherapy is a first-line globe-sparing therapy for retinoblastoma and can be delivered through intravenous, intraarterial, sub-Tenon fascia, and intravitreal routes [7]. IAC has assumed a major role in retinoblastoma management, achieving globe salvage and resolution of retinal detachment with minimal systemic toxicities [8, 9]. Studies have documented that IAC can be employed as a primary treatment or secondary (rescue) treatment for eyes that fail alternative therapies [10]. Overall, IAC achieves retinoblastoma control with globe salvage in 72% of eyes as a primary therapy and 62% of eyes when used as secondary therapy [10]. Our patient had initial treatment with intravenous chemotherapy and was secondarily treated with IAC, achieving immediate tumor control, and with foveolar preservation, despite the proximity of the tumor within 615 µm to the foveola.

Imaging of retinoblastoma includes digital wide-field fundus photography, fluorescein angiography, ultrasonography, and now HH-OCT. In this case we used the stand-mounted iVue Optovue (Optovue, Freemont, CA) to capture the tumor. The iVue provides up to 21° field of view and can be mounted on a wheeled stand which offers stability when acquiring OCT images during examination under anesthesia. HH-OCT scans using iVue can be captured with minimal pupillary dilation but a fully dilated pupil yields better results. HH-OCT provides precise submillimeter tumor documentation within the retina and even into the choroid [4, 11, 12]. In fact, HH-OCT can provide detection of “invisible” retinoblastoma, first reported by Saktanasate et al. in which a subclinical tumor was coincidentally captured on HH-OCT in the macular region, and localized to the outer nuclear layer [4]. Berry et al. later demonstrated the use of SD-OCT to identify subclinical retinoblastoma, estimating that this technology could allow earlier diagnosis and treatment with less treatment-related damage to ocular structures and more careful surveillance [11]. Soliman et al. evaluated the use of HH-OCT in 63 eyes and found that in 339 HH-OCT sessions 92% were informative, of which OCT directed diagnosis in 16% and treatment in 58% [13]. They also noted that OCT influenced pre-OCT treatment plans in 15% of all OCT sessions. In this case, we confirmed clinical suspicion of recurrence using HH-OCT documenting a homogenous dome-shaped intraretinal mass with inner retinal draping over the solid tumor. We subsequently documented complete tumor response to IAC with OCT-evidence of concave regression and preservation of the foveola, despite the parafoveolar tumor location.

In our case, tumor recurrence was detected early with HH-OCT, allowing intervention with IAC for complete tumor regression and preservation of the foveal microanatomy. In most cases, 3 cycles of IAC are necessary for tumor control, but given the submillimeter recurrence, only 1 cycle was necessary to achieve control with no further recurrence on 2-year follow-up.

In summary, HH-OCT is an invaluable tool in retinoblastoma management, providing information that aids treatment decisions, particularly in juxtafoveal tumors. The role of HH-OCT in retinoblastoma management includes detection of subclinical new tumors, monitoring of tumor regression, detection of small tumor recurrence, and judgment of foveolar microanatomy with estimation of visual potential in a preverbal child.

## Abbreviations

OD: Right eye
OS: Left eye
OU: both eyes
OCT: Optical coherence tomography
SD-OCT: Spectral-domain optical coherence tomography
HH-OCT: Handheld optical coherence tomography
IAC: Intraarterial chemotherapy
CRD: Chemoreduction
